# An Ensemble Approach for Robust Automated Crack Detection and Segmentation in Concrete Structures

**DOI:** 10.3390/s24010257

**Published:** 2024-01-01

**Authors:** Muhammad Sohaib, Saima Jamil, Jong-Myon Kim

**Affiliations:** 1 School of Computer Science and Technology, Zhejiang Normal University, Jinhua 321004, China; sohaibdurrani@zjnu.edu.cn; 2 Zhejiang Institute of Photoelectronics & Zhejiang Institute for Advanced Light Source, Zhejiang Normal University, Jinhua 321004, China; 3 Department of Computer Science, Virtual University of Pakistan, Peshawar 25000, Pakistan; saima.jamil@vu.edu.pk; 4 Department of Electrical, Electronic and Computer Engineering, University of Ulsan, Ulsan 44610, Republic of Korea; 5 Prognosis and Diagnostics Technologies Co., Ltd., Ulsan 44610, Republic of Korea

**Keywords:** crack detection, concrete structures, structural health monitoring, ensemble YOLOv8

## Abstract

To prevent potential instability the early detection of cracks is imperative due to the prevalent use of concrete in critical infrastructure. Automated techniques leveraging artificial intelligence, machine learning, and deep learning as the traditional manual inspection methods are time-consuming. The existing automated concrete crack detection algorithms, despite recent advancements, face challenges in robustness, particularly in precise crack detection amidst complex backgrounds and visual distractions, while also maintaining low inference times. Therefore, this paper introduces a novel ensemble mechanism based on multiple quantized You Only Look Once version 8 (YOLOv8) models for the detection and segmentation of cracks in concrete structures. The proposed model is tested on different concrete crack datasets yielding enhanced segmentation results with at least 89.62% precision and intersection over a union score of 0.88. Moreover, the inference time per image is reduced to 27 milliseconds which is at least a 5% improvement over other models in the comparison. This is achieved by amalgamating the predictions of the trained models to calculate the final segmentation mask. The noteworthy contributions of this work encompass the creation of a model with low inference time, an ensemble mechanism for robust crack segmentation, and the enhancement of the learning capabilities of crack detection models. The fast inference time of the model renders it appropriate for real-time applications, effectively tackling challenges in infrastructure maintenance and safety.

## 1. Introduction

Concrete is an extensively used material in building infrastructures, including bridges, buildings, roads, and pavements. Nevertheless, the structural integrity of concrete constructions undergoes a natural degradation process due to several factors. These factors include environmental impacts, excessive loads, and the slow deterioration of components [1,2,3]. The identification of cracks in concrete structures has great significance. These cracks indicate the early stages of degradation, presenting a substantial threat to the durability and stability of the structure [4]. Cracks function as entry points, facilitating the ingress of water and deleterious chemicals in concrete structures. As a consequence, issues such as corrosion of reinforcing bars (rebar), disintegration, and spalling in the structures arise [5,6]. These concerns have the potential to significantly undermine public safety and endanger the structural integrity of the construction project.

Historically, the conventional practice for detecting these fissures has used manual visual examinations [7]. Nevertheless, this approach is not without its difficulties as it is characterized by a significant investment of time, a large amount of effort, and a strong dependence on the inspector’s competence. Moreover, the practice of human inspections is accompanied by inherent safety hazards [8]. Therefore, non-invasive methods for assessing the health of concrete structures have been developed and studied in the literature which are becoming increasingly essential in the management of smart facilities [8]. These systems are based on data-driven artificial intelligence (AI) techniques in which data are usually collected through magnetic shape memory alloys (MSMA), capacitive sensors, embedded piezoelectric (PZT) sensors, and digital cameras [9,10,11]. Automated procedures provide a more economically advantageous, streamlined, and secure substitute for human inspections. The use of machine learning and digital image processing methods for crack detection has been a notable focus of study in this field [12].

It is effective and robust in detecting cracks in concrete structures through the segmentation of digital images. In this perspective, Bhattacharya et al. proposed an interleaved deep artifacts-aware attention mechanism (iDAAM) to classify images containing structural faults [13]. The algorithm could extract local discriminant features benefiting the defect identification in the images. In another study, Zhao et al. [14] proposed a feature pyramid network (crack-FPN) for crack detection, segmentation, and width estimation. First crack detection was performed through You Only Look Once version 5 (YOLOv5) model, and it was later segmented by crack-FPN. Although the proposed algorithm could effectively detect and segment the crack, the proposed methodology had a relatively greater inference time on the test images. Similarly, Zhang et al. [15] presented a MobileNetv3-based broad learning-based effective concrete surface cracks detection mechanism with high accuracy and improved learning time. In this work, first, features were extracted from the images through MobileNetv3 which were later mapped in a broad learning system to identify cracks. A binary convolutional neural network (CNN) was presented for the identification of cracks in concrete structures [16]. It integrated regression models such as random forest (RF) and XGBoost and exhibited a high accuracy on a publicly available dataset. However, the accuracy of the model deteriorated when tested with unseen data. In [17] an optimized Deeplabv3+ BDF network is proposed for concrete crack segmentation. The network is trained using transfer learning, coarse-annotation, and fine-annotation techniques. The developed model could effectively detect cracks in the images.

Although numerous studies have been published on this topic exhibiting satisfactory crack inference performance on images with non-complex backgrounds. Nevertheless, a significant variation in their performance can be observed when tested on images with complex backgrounds, i.e., in the presence of many visual distractions, challenging illumination conditions, and complex backgrounds. Moreover, a lower inference rate is another concern that is associated with the available strategies in the literature. A crack detection model shall be easily adoptable in real-time applications and scalable with lower inference time for test images, ensuring high precision, accuracy, and robustness.

This work presents a novel approach to effectively infer and segment cracks in images with complex backgrounds and distribution patterns with high generalization power. Furthermore, the proposed model is capable of inferring cracks in less time with high precession. The proposed approach introduces an ensemble approach for YOLOv8 models. Using this approach, first, abstract characteristics useful for crack detection are derived from images containing cracks using three YOLOv8 models, i.e., YOLOv8x, YOLOv8m, and YOLOv8s models. Later, the trained models are quantized and combined to create an ensemble of YOLOv8 models in the inference stage. The final prediction of this approach is determined by concatenating the outputs of the trained models. This ensemble approach provides better segmentation results with higher intersection over union (IoU) and confidence threshold values. Moreover, the inference time for the ensemble model on the test images is quite low which makes the proposed model easily adaptable in real-time scenarios with a potential of scalability. The three main contributions of this work are given below.

1. The main contribution is the improve the crack segmentation capability of the YOLOv8 model from the inferred results.
2. Furthermore, it demonstrates that the inference process of a YOLOv8 model may be accelerated by quantizing the model, which will be advantageous for the real-time implementation of the suggested model.
3. Lastly, it introduces an ensemble technique to combine the inference outcomes of many YOLOv8 models in order to enhance the ultimate segmentation results.

The rest of the paper is structured as follows: Section 2 offers a detailed examination of the technical background, encompassing a thorough description of the methodologies that underpin the research. Section 3 presents a thorough elucidation of the adopted methodology. In Section 4, the description of the dataset used in this study is presented to ensure transparency and capacity to be replicated. In Section 5 the hyperparameters tuning process for the proposed model is explained. Section 6 discusses the results, accompanied by a comprehensive analysis, which offers valuable insights into the efficacy of the suggested methodology. The last section, i.e., Section 7, summarizes the whole work. 

## 2. Technical Background

To mitigate additional damage to concrete structures and uphold public safety, the automated assessment of concrete structures is of utmost importance. This study endeavors to deliver an automated mechanism for detecting cracks, emphasizing efficiency, rationality, and precision in the overall process and outcomes. To construct a comprehensive framework endowed with high generalization power for crack detection, we explore the application of the deep learning technique YOLOv8, specifically delving into the ensemble of quantized YOLOv8 models. The technical intricacies of these methods are expounded upon in the proceeding subsections.

### 2.1. Overview of the YOLOv8 Model

**Figure 1:**
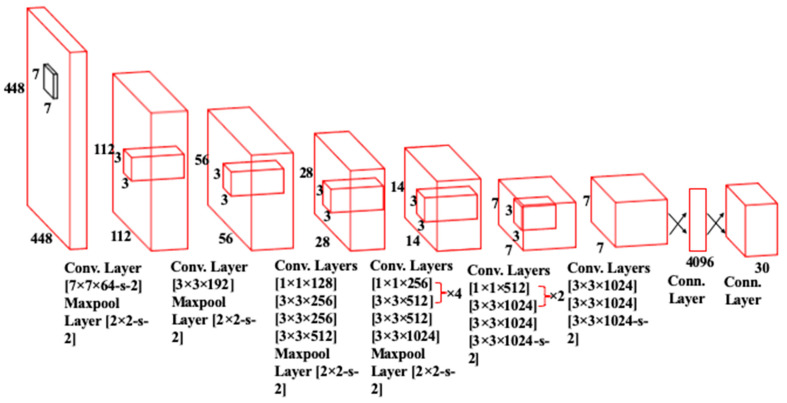
The architecture of YOLOv1 Network [20].

**Figure 2:**
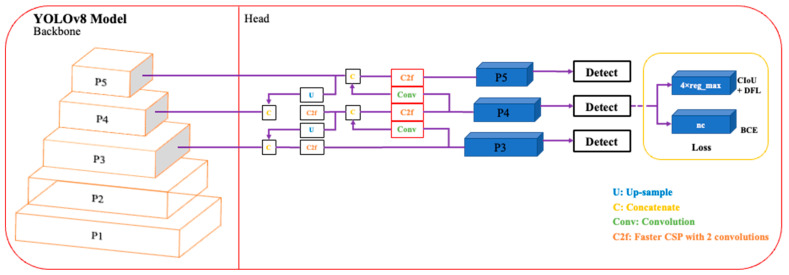
An illustration of YOLOv8 Architecture [25].

The basic structure of the You Only Look Once (YOLOv1) network as described in [18] is given in Figure 1. It contains a total of 26 layers of which 24 are convolutional layers and 2 are fully connected layers. The convolution layers are used to extract feature maps from the inputs. The extracted feature maps through convolution operations are down-sampled to reduce their dimensions. The output of the network is a 7 × 7 × 30 tensor. Moreover, it uses stochastic gradient descent as an optimizer. Over the years, different variants of the YOLO network have been released with YOLOv8 the latest member of the YOLO series. The schematics of this latest variant are presented in Figure 2. This current iteration maintains the same architectural structure as its previous versions, namely version 6 [19]. However, it incorporates some enhancements in comparison to other versions of YOLO, i.e., it integrates the feature pyramid network (FPN) with the path aggregation network (PAN). Moreover, it also has an updated image annotation mechanism including automated labeling, shortcuts to perform labeling efficiently, and hotkeys that facilitate the training of a model. The FPN module steadily decreases the spatial resolution of the inputs while instantaneously increasing the number of channels for the feature. In this way, it forms feature maps that can identify objects of varying scales and resolutions. In contrast, the PAN module combines features from different layers of the network by using skip connections. This technique is helpful for the model to explore features at various scales and resolutions, hence, benefiting the model to identify objects with diverse dimensions and configurations [20]. The complete architecture of YOLOv8 is discussed in the following subsections.

#### 2.1.1. Backbone Network

In YOLOv8 the backbone network consists of a customized CSPDarknet53 network [21], in which inputs are first down-sampled five times, resulting in five distinct scales of features. In the updated structure, the backbone network uses a C2f module, i.e., a faster cross-stage partial (CSP) bottleneck with two convolutions instead of traditional CSP. In the C2f module, the information flow is optimized through a gradient shunt connection. It provides a richer flow of gradients within the architecture, hence, reducing the computational complexity and also lightweight network design. First, convolution and batch normalization operations are performed on the inputs, and later, the output of the network is obtained by activating the information stream using a sigmoid-weighted linear unit (SiLU). In YOLOv8 the spatial pyramid pooling fast (SPPF) is used to generate feature maps of constant size at the input, and adjustable dimensions at the output. Moreover, it also effectively reduces computational complexity and latency by linking the three highest pooling levels [22], as compared to the SPPF.

#### 2.1.2. Neck Module

The neck part of YOLOv8 is inspired by the PANet architecture [23] and incorporates a Path Aggregation Network and Feature Pyramid Network (PAN-FPN) arrangement. In contrast to YOLOv5 and YOLOv6, the convolutional (conv) step that follows the up-sampling (U) of the PAN module is absent in YOLOv8 which results in a more efficient and lightweight model. The PAN-FPN architecture unifies top-down and bottom-up approaches that concatenate (C) the shallow and deep semantic information resulting in diverse and comprehensive features.

#### 2.1.3. Head Module

The detection module of YOLOv8 applies a decoupled head structure where there is a separate branch for classification and predicted bounding box regression. This detection structure helps in object detection with high precision and accelerates the convergence of the model. Moreover, YOLOv8 relies on an anchor-free approach for the detection module that effectively identifies positive and negative samples. In addition, to improve detection accuracy and resilience, it incorporates the Task-Aligned Assigner [24] to dynamically allocate samples.

### 2.2. Loss Calculation

YOLOv8 uses Complete Intersection over Union (CIoU) and Distributional Focal Loss (DFL) [26,27] to calculate the regression loss of the bounding boxes. The CIoU takes into aspect ratio of the ground truth and predicted bounding boxes and uses an inverse trigonometric function to minimize the difference between the two entities. The mathematical expression of the CIoU loss, i.e., $Loss_{CIoU}$ is given below [26]:

(1) $$Loss_{CIoU}=1-IoU+\frac{D^{2}\left(b,b^{GT}\right)}{{\left(MB_{w}\right)}^{2}+{\left(MB_{h}\right)}^{2}}+\frac{4}{\pi^{2}}\left(\tan^{-1}\frac{w^{GT}}{h^{GT}}-\tan^{-1}\frac{w}{h}\right)$$

where, IoU is the notion used for intersection over union which represents the intersection ratio between the predicted and true bounding boxes. Moreover, $D\left(b,b^{GT}\right)$ represents the Euclidean distance between the predicted and the real boxes; the height and width of the predicted box are denoted by h and w, whereas, $h^{GT}$ and $w^{GT}$ denote the height and width of the ground truth bounding box. Similarly, $MB_{w}$ and $MB_{h}$ identify the width and height of the minimum box that encloses the prediction and true boxes.

In addition to the CIoU YOlOV8 also takes advantage of the DFL to rapidly regress over the values near a label L by enlarging the probabilities for $L_{i}$ and $L_{i+1}$. As a result, it increases the optimization efficiency of the model by predicting the label with high confidence. The expression of the DFL is given as follows [27].

(2) $$DFL\left(P\left(L_{i}\right),P\left(L_{i+1}\right)\right)=\left(\left(L_{i+1}-L\right)\;\log P\left(L_{i}\right)+\left(L-L_{i}\right)\log P_{L+1}\right)$$

In this equation, $P\left(L_{i}\right)$ and $P\left(L_{i+1}\right)$ represent the distribution of labels $L_{i}$ and $L_{i+1}$.

## 3. Methodology

**Figure 3:**
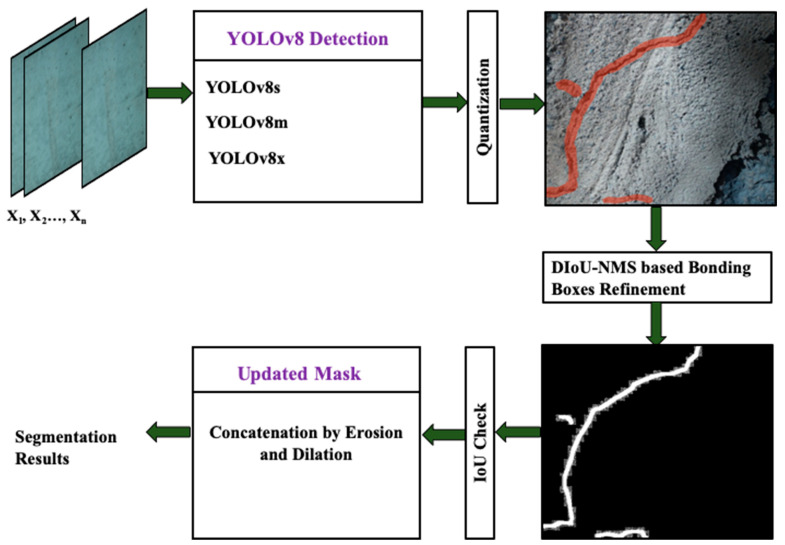
The illustration of the proposed ensemble YOLOv8 for the automatic detection of surface cracks in concrete structures.

This study presents a robust crack detection and segmentation mechanism by employing an ensemble of quantized YOLOv8 models. The methodology to segment cracks comprises three discrete steps, i.e., training and quantization of the models, inference of cracks, and segmentation of the inferred cracks, as depicted in Figure 3.

### 3.1. Training and Quantization

In this work, three YOLOv8 models, including YOLOv8s, YOLOv8m, and YOLOv8x, are utilized to create an ensemble model. The suffixes s, m, and x stand for small, medium, and extra-large sizes of the models. The size of the model represents the number of learnable parameters that each model contains. So, YOLOv8s, YOLOv8m, and YOLOv8x have 11.8, 27.3, and 71.8 million parameters, respectively. First, each model is trained on a distinct image subset. Training with distinct subsets ensures exposure to diverse images of concrete structures with cracks. It also prevents overfitting by enabling each model to learn distinct abstract characteristics. After training, the models are quantized before the inference of cracks on the unseen dataset. The quantization process includes reducing the precision of the weights and activation of the models. It helps the models to utilize less memory, achieve higher inference speed, and reduce latency.

### 3.2. Inference

Following the training process, every model is subsequently tested on unseen images to infer cracks. During inference, non-maximum suppression (NMS) is implemented by applying given thresholds, i.e., IoU and confidence value both set to 0.5. The YOLOv8 model utilizes distance-based intersection over union NMS (DIoU-NMS) to eliminate bounding boxes with low confidence and repetitive findings.

### 3.3. Segmentation

**Figure 4:**
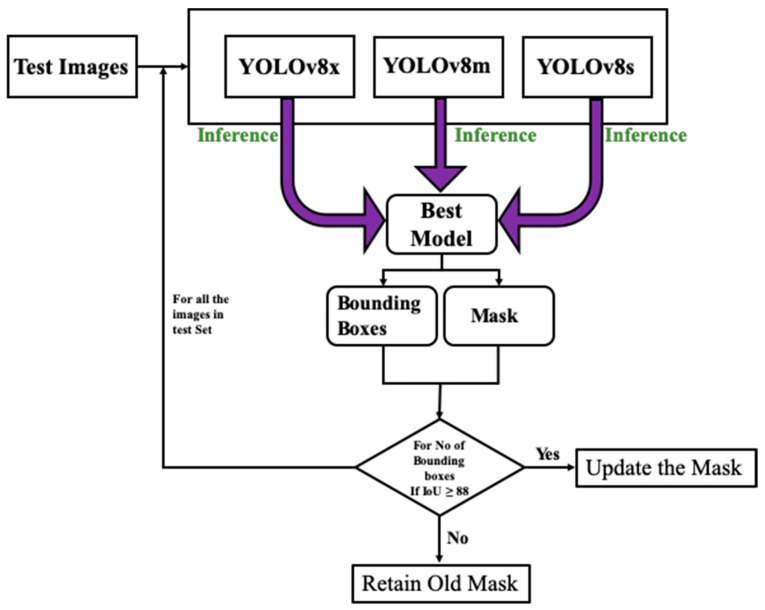
The illustration of the segmentation mask refinement process.

During the post-inference stage, ensemble predictions are generated by amalgamating outcomes from the three models. Ensemble prediction enhances object detection and segmentation masks to achieve higher levels of accuracy. Refinement entails the evaluation of mask overlap by employing the intersection over union (IoU) concept as illustrated in Figure 4. The mask and bounding box of the top predictor function as a point of reference for the refinement procedure. If the Intersection over Union between the masks of the top-performing and second-best models exceeds 88% for all bounding boxes, apply morphological operations (namely, erosion and dilation) to update the mask. Otherwise, keep the original mask unchanged. The identical procedure is iterated using the outcomes of the previous model. The refinement method is implemented on every image in the test dataset to achieve the ultimate segmentation outcomes.

By organizing the methodology into three essential stages, our goal is to improve clarity and promote a more comprehensive comprehension of the suggested deep learning technique for crack identification and segmentation.

In Equation (5) AP is the average precision of the category with index value i and the number of categories N which in this case is 2.

## 4. Dataset Description and Configuration

**Figure 5:**
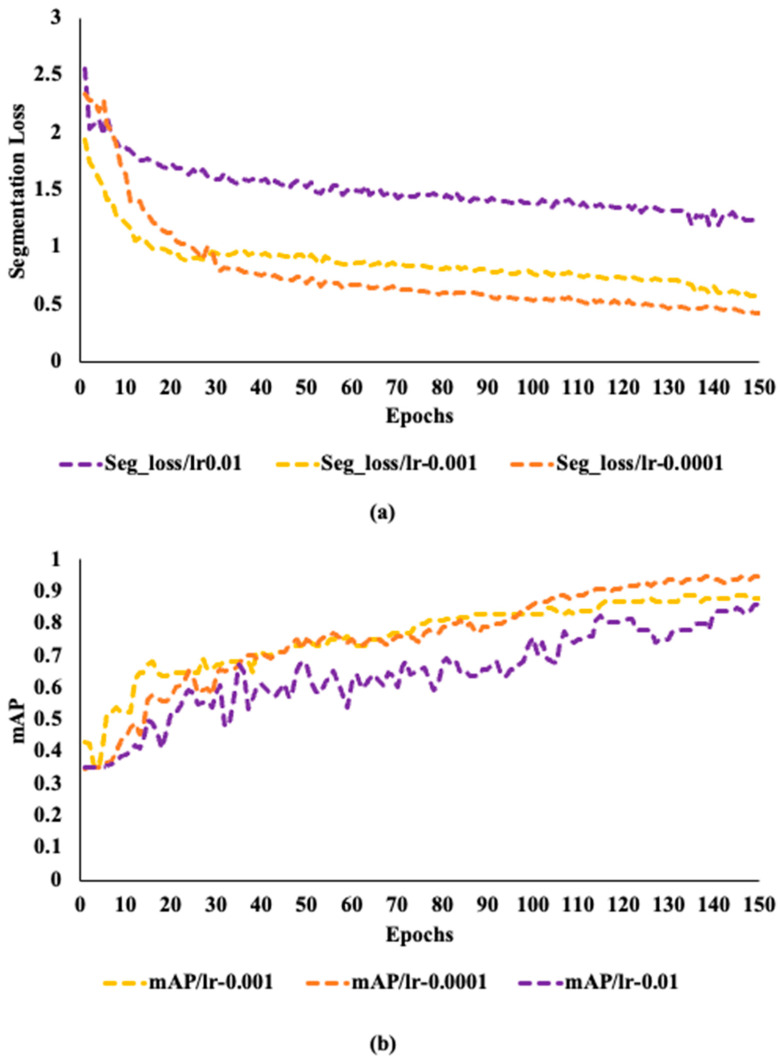
The performance matrices of the model: (**a**) the segmentation loss of the model under different fixed learning rates, (**b**) the mean average precision (mAP) of the model under different fixed learning rates.

In this study, a comprehensive dataset for crack segmentation consisting of 6315 crack images with labels was used to develop and evaluate the proposed model [28]. The dataset contains images of cracks in a range of constructions, such as buildings, bridges, and roadways with 450 × 450 resolution. Out of all the images, 75% of the images were used to train the three YOLOv8 models. The remaining images are used as inference data to evaluate the model performance. The images selected for the training were further divided into three subsets to create separate training sets for the three models. The configuration details for the dataset during the experiment are presented in Table 1 along with samples illustrated in Figure 5.

## 5. Development and Evaluation of the Model

The training of a neural network is important to ensure that the developed model has updated its parameters according to the task at hand. The initial weights are obtained from three pre-trained YOLOv8 models on the ImageNet dataset. The training data undergoes several transformations such as flipping, rotating, scaling, and other transformations to enhance its diversity. The models are developed using the data configuration discussed in Section 4, i.e., each model is trained using one of the training subsets and is evaluated on the test subset. Moreover, a batch size of 64 and an Adam optimizer are used to train the network. The performance of a network is mostly subjected to key hyperparameters such as the learning rate and the number of epochs used to train the network.

In order to find out the optimal hyperparameter setting, initially, YOLOv8s is trained using learning rates of 0.01, 0.001, and 0.0001, and 50, 100, 150, 200, and 300 epochs. Afterward, all three models are trained using the optimal configuration for hyperparameters explored earlier.

### 5.1. Hyper Parameters Tuning

Figure 5 shows how segmentation loss and mAP for the three models vary over the epochs when training at various fixed learning rates. Figure 5a shows that throughout training, the segmentation loss first drops off quickly before steadily stabilizing as the number of epochs rises. On the contrary, mAP has a different behavior as depicted in Figure 5b. Better training performance is ensured when the learning rate is 0.0001 than the other two learning rates, as the mAP rises gradually with few changes to a value of 0.91.

**Figure 6:**
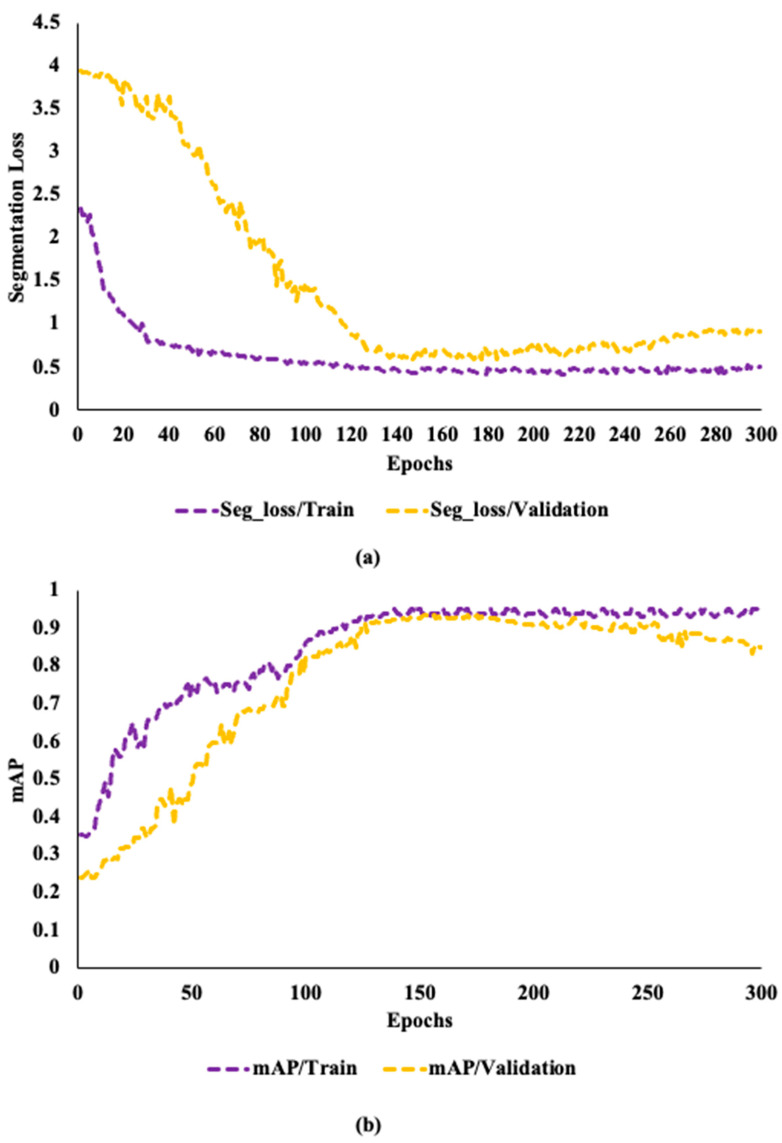
The performance matrices of the model: (**a**) the train and validation segmentation loss of the model for different epochs, (**b**) the training and validation mean average precision (mAP) of the model under different epochs.

To determine the overfitting, the model was trained with 50, 100, 150, 200, and 300 epochs. The changes in segmentation loss and mAP for the network trained on the dataset with a learning rate of 0.0001 reach a limit after 150 epochs. The network in training and validation sets retains a good learning efficiency for the first 150 epochs. Even while the segmentation loss and mAP demonstrate improvement after 150 epochs, this performance could point to overfitting. Due to the parameters showing a steadily reduced network learning on the validation set. Figure 6a,b shows that over the next 150 epochs, segmentation loss only drops by 0.004 and mAP only grows by 0.005. Due to high efficiency, this study employs 150 epochs for training all the YOLOv8 models at a learning rate of 0.0001, and grayscale images.

### 5.2. Performance Evaluation Matrices

The precision, recall, mAP0.5, and inference speed are used as evaluation matrices to assess the object detection performance of the proposed ensemble Yolov8 model. The mathematical formulation to calculate the precession of a model is given below.

(3) $$Precision=\frac{True\;positive}{True\;positive+False\;Positive}$$

Moreover, the ratio of accurately predicted true positive samples by the model to the number of actual true positive samples is known as recall. The mathematical expression for the recall calculation is as follows.

(4) $$\frac{True\;Positive}{True\;Positive+False\;Negative}$$

Furthermore, Equation (5) illustrates the formula for calculating mean average precision (mAP), which is the outcome of a weighted average of the average precision values of all sample categories. This metric is used to assess the detection ability of the model across all categories.

(5) $$mAP=\frac{1}{N}\overset{N}{\underset{i=1}{\sum }}AP_{i}$$

## 6. Result Analysis

To build a robust framework for crack detection, this study employs deep learning techniques, consisting of an ensemble of the YOLOv8 models, i.e., YOLOv8x, YOLOv8m, and YOLOv8s. The overall methodology consists of three phases, (1) the training and quantization of the three models, (2) the inference of the cracks in the test images, and (3) the extraction of the masks for cracks from the inferred results through the segmentation process. The criteria to present the final segmented results is to concatenate the masks of the three models based on the calculation of IoU among the masks of the three masks models. The results presented here are generated on a Tesla T4 GPU with 16 GB of random access memory (RAM). In Table 2, the precision, recall, and mAP for the three models during the training and validation phases are presented. It can be observed that during the training phase, the YOLOv8x has the highest precision, recall, and mAP, i.e., 93.13%, 91%, and 90.13%, respectively. The second-best performance is of the YOLOv8m with 92.20%, 90.68%, and 89.90%, precision, recall, and mAP values, respectively. The least precision, recall, and mAP values can be observed for the YOLOv8s model with 90.69%, 89.53%, and 88.24%, respectively. A similar trend is observed in the case of the validation phase of the models. The maximum values for precision and recall matrices are observed for the YOLOv8x model followed by YOLOv8m, and YOLOv8s. The convergence of all the models towards optimal values in both the training and validation phases indicates that the models are ready to be evaluated on the unseen data and make the inference about the cracks.

**Figure 7:**
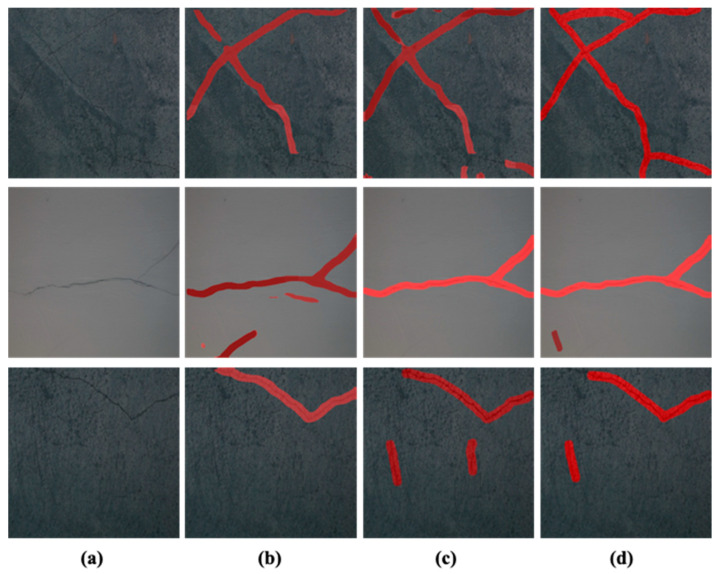
The original images and the inference results: (**a**) the original images, (**b**) the inference result for YOLOv8x, (**c**) the inference results for YOLOv8m, and (**d**) the inference results for YOLOv8s.

The models are evaluated on the test dataset containing 1578 images as discussed in Section 4. In Figure 7, the original images and the inference results from the three models are given in Figure 7a–d. It is evident from the inferred results that all the models could infer cracks in the test images. However, the inference results of the three models cover the crack area with varying confidence levels. It can be YOLOv8x accurately identifying cracks as evident in Figure 7b. Similarly, in Figure 7c it is noticeable that the YOLOv8m model identification result is better than the rest of the two models, and in Figure 7d the superiority of the YOLOv8s is evident. It is noteworthy that the inference performance of the models is not the same for all the images. It can be assumed that the three models have acquired different knowledge from the datasets during the training phase. Therefore, the behavior of the three models differs during the inference phase. Due to this reason, an ensemble segmentation approach based on the result of the three YOLOv8 models is adopted to refine the final segmentation results.

**Figure 8:**
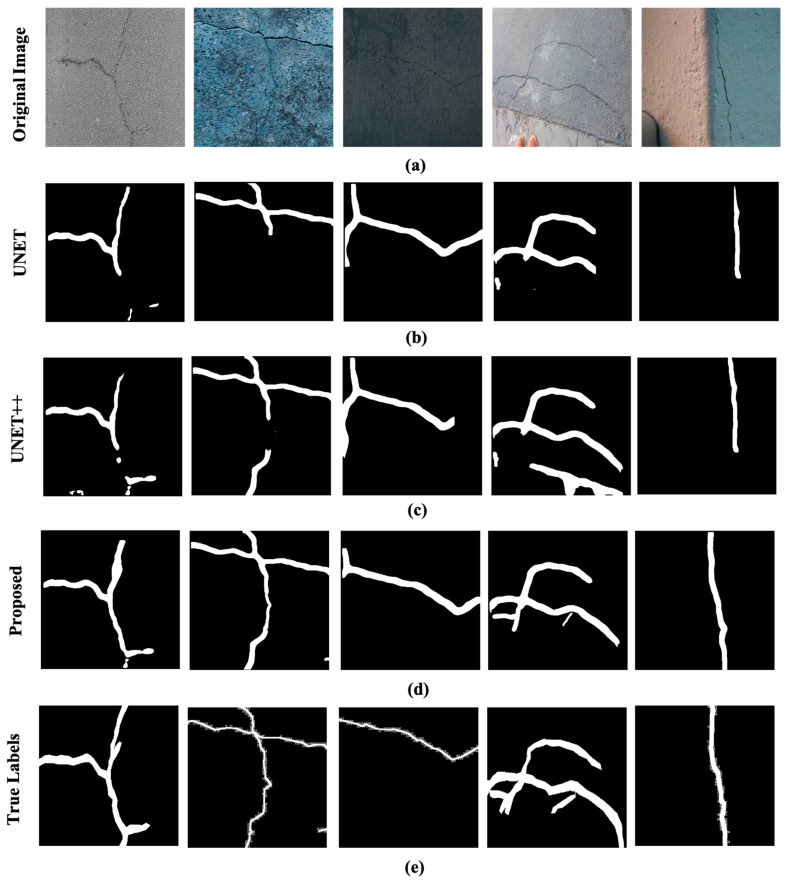
The results on the heterogenous dataset: (**a**) the original images, (**b**) the segmentation masks generated through UNET, (**c**) the segmentation masks generated through UNET++, (**d**) the segmentation masks generated through the propped model, (**e**) and the true labels.

In Figure 8, a few of the original images in the test dataset, the segmentation masks generated through various models as the true labels are presented. The original images given in Figure 8a contain complex backgrounds and varying illumination conditions which makes the segmentation task more challenging. It is apparent in Figure 8d, that the segmentation masks generated through the proposed approach depict a close resemblance to that of the true label for the given images shown in Figure 8e. Moreover, the segmentation results of the proposed model are compared with a few state-of-the-art crack models, i.e., deep U shaped Network (U-NET) and U-NET++ used for crack detection as shown in Figure 8c,d. The segmentation performance of the UNET and U-NET++ in the presence of a challenging background and illumination deteriorates as evident in the pictures. Based on the segmentation results presented in Figure 8, the superiority of the proposed ensemble approach to segment the cracks is justified.

### 6.1. Checking the Generalization Performance

**Figure 9:**
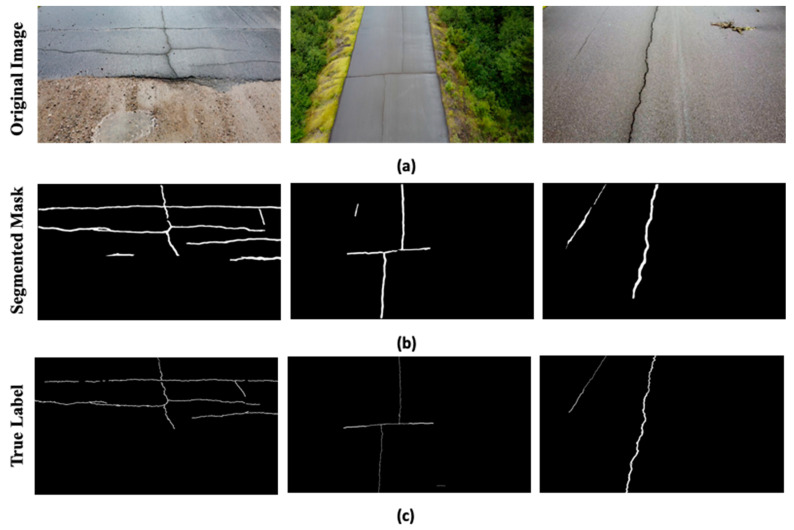
The results on the pavement cracks dataset: (**a**) the original images, (**b**) the segmentation masks generated through the proposed model, (**c**) and the true labels.

**Figure 10:**
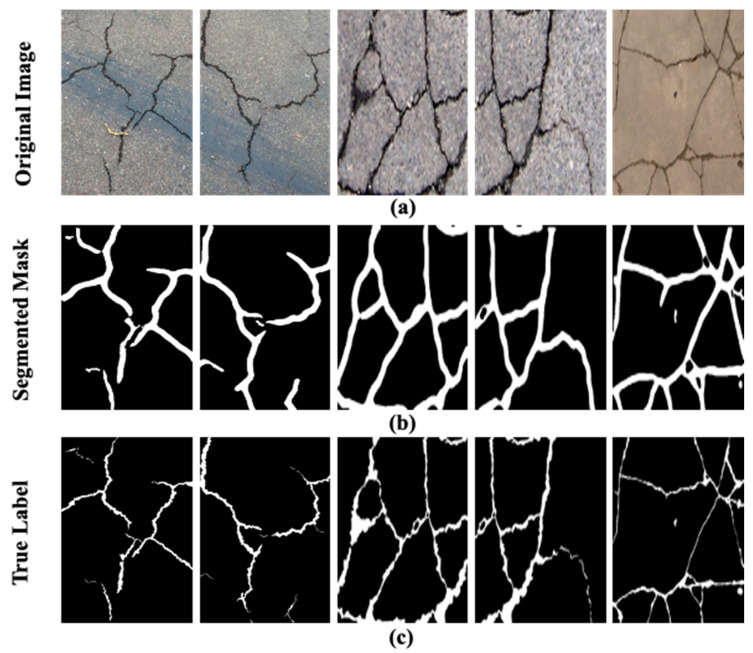
The results on the deepcrack dataset: (**a**) the original images, (**b**) the segmentation masks generated through the proposed model, (**c**) and the true labels.

To check the generalization performance, the proposed model is further evaluated on the pavement cracks [29] and deepcrack datasets [30]. The pavement cracks dataset contains images of a 386 m long road taken with a 12-megapixel camera, an 83-degree field of view, and 1920 × 1080 resolution. Similarly, the deepcrack dataset contains 537 images of concrete structures with 544 × 384 resolution. It is challenging to perform the crack detection and segmentation of the drone images as it contains extra information in the scene such as greenery, trees, and edges of the road. The presence of the extra information adds complexity to the ground region. Moreover, the irregular pattern of cracks (diagonal and crazing) in various scenes and scales as given in deepcrack dataset also makes the segmentation a challenging task. The qualitative results of the proposed model on the pavement cracks and deepcrack datasets segmentation are shown in Figure 9a–c and Figure 10a–c, respectively. It can be perceived from the segmentation results in Figure 9b while using the pavement cracks dataset that the proposed ensemble YOLOv8 could infer the cracks in these complex images with high quality. However, during segmentation, it also included outliers such as edges of the road as cracks, as the extra information present in the images adds complexity to the segmentation process. Nevertheless, overall segmentation results presented in the figure closely resemble the true labels given in Figure 9c. It is evident from the segmentation results given in Figure 10b that the proposed model could also segment cracks with complex skeleton and distribution patterns. These segmented masks are similar to the true labels given in Figure 10c validating the efficacy of the proposed model. These results justify that the proposed model has high generalization power as it could infer cracks in the images with different resolutions and more complex backgrounds.

To further consolidate the analysis a comprehensive analysis in terms of precision, recall, and F1 score for the proposed model in contrast to existing state-of-the-art techniques is given in Table 3. These metrics are essential for evaluating the performance of the model. The results are generated by utilizing three distinct datasets, i.e., the heterogeneous dataset [28], the pavement cracks dataset [29], and the deepcrack dataset [30].

In the case of the heterogeneous dataset, the proposed model consistently demonstrated superior performance in comparison to the U-NET and U-NET++ models across all the assessment metrics. There is an enhancement of at least 3.88%, 3.68%, and 3.78% in precision, recall, and F1 score for the proposed model. It is noteworthy that the proposed model is capable of yielding results with high precision in comparatively less inference time, i.e., 2 milliseconds less time per image. The inference time is a critical factor, particularly in situations that necessitate quick decision-making or processing large volumes of images in realtime.

In the case of the pavement cracks and deepcrack dataset, the superiority of the proposed model is once again evident. It has the highest values for precision, recall, and F1 score as well as high inference speed. The results of the proposed model are at least 5.36%, more precise than the other two networks on both datasets. Similarly, at least 6.82% and 6.11% improvement can be observed for recall and F1 score, respectively. The inference results are at least 2 milliseconds faster on both datasets. The fact that the suggested model consistently outperforms the comparison models highlights its ability to effectively infer cracks in different datasets, confirming its generalization ability.

### 6.2. Ablation Analysis

In this section, we evaluated the performance impact of the different modules of the suggested strategy by an ablation analysis. To assess the relevant changes in performance metrics, we specifically examined quantization and ensemble prediction modules and removed them one at a time with the dataset containing heterogeneous images [30]. The complete description of the ablation analysis is presented in Table 4.

Following the comprehensive ablation study, it is evident that the IoU threshold for the three models significantly diminishes in the absence of the ensemble module. The deterioration occurs because the inference of each model on the test image varies. This variation is based on the specific set of abstract features acquired by each model during the training phase. Furthermore, the inference time of individual models on a single image is shorter compared to the ensemble model. In general, the inclusion of the ensemble YOLOv8 model has resulted in a significant enhancement in segmentation performance, as seen by a 3% rise. This improvement emphasizes the extent to which one model in the ensemble enhances the others, leading to a segmentation that is more dependable and precise.

Moreover, an analysis was conducted to assess the impact of quantization on the segmentation performance of the model. Curiously, the data shows that quantization does not have a direct impact on segmentation performance. Conversely, it has a substantial role in a 7% decrease in computational expenses, as evidenced by the table.

## 7. Conclusions

This method introduces a dual strategy that leverages the advantages of quantization and YOLOv8 to enhance the precision and efficiency of concrete crack detection and segmentation. To enhance the efficiency of the process and optimize resource usage, the proposed method incorporates an ensemble approach of multiple quantized YOLOv8 models. First, three variants of YOLOv8 models are trained on three distinct subsets of concrete crack images. Afterward, the trained models are quantized to improve the inference speed of the models. Later, for better segmentation of cracks, the proposed approach concatenates the inference results of multiple quantized YOLOv8 models based on the IoU thresholding criterion. The quantization of the models expedites the inference process, which involves reducing the precision of model weights and activations. Moreover, the ensemble model improves the learning capability of crack detection models by first extracting abstract characteristics through different pre-trained quantized models. From the results of the different experiments, it is evident that the proposed model could infer and segment cracks in the images containing complex backgrounds. The proposed demonstrated enhanced segmentation results with at least 89.62% precision and an intersection over union score of 0.88. Moreover, the inference time per image is reduced to 27 milliseconds which is at least a 5% improvement over other models in the comparison. In summary, this paper introduces a new technique that integrates quantization approaches with the YOLOv8 segmentation paradigm. The proposed model is highly suitable for real-time applications due to the integration of quantized YOLOv8 models. This integration enhances crack detection performance, mitigates overfitting, and optimizes inference time.

## Figures and Tables

**Table 1:** A description of the organization of the data for training and testing phases.

Total no. of Samples in the Dataset	Details of the Data Samples		
4215	Datasets	Training (75%)	Test (25%)
	Dataset-1	1054	1053
	Dataset-2	1054	
	Dataset-3	1054	

**Table 2:** The performance matrices for the three models during the training and validation phases.

Model	Train			Validation		
	Precision (%)	Recall (%)	mAP (%)	Precision (%)	Recall (%)	mAP (%)
YOLOv8x	93.13	91	90.13	92.33	90.02	89.50
YOLOv8m	92.20	90.68	89.90	91.40	89.05	88.13
YOLOv8s	90.69	89.53	88.24	89.01	86.97	86.5

**Table 3:** The comparison of the performance matrices for the proposed model and other state-of-the-art models.

Heterogenous Dataset				
Method	Precision	Recall	F1	Inference (Milliseconds/Image)
U-NET	84.18	82.09	83.12	29
U-NET++	87.03	85	86	30
Proposed	90.91	88.68	89.78	27
**Pavement Cracks Dataset**				
U-NET	81.08	79.14	80.10	35
U-NET++	83.63	80.29	81.93	37
Proposed	89.62	87.91	88.76	30
**Deepcrack Dataset**				
U-NET	81.76	80.01	80.88	27
U-NET++	84.57	81.14	82.82	28
Proposed	89.93	87.96	88.93	25

**Table 4:** The performance matrices with and without the ensemble technique and quantization.

Ablation Setting	IoU Threshold	Precision (%)	Recall	Inference Time (Millisecond)
YOLOv8s (w/o Ensemble and quantization) YOLOv8m (w/o Ensemble and quantization) YOLOv8x (w/o Ensemble and quantization)	0.50	93.13	91	12
	0.50	92.20	90.68	18
	0.50	89.01	89.53	32
Ensemble Technique (w/o quantization)	0.88	91.44	90.40	34
Quantization	0.88	90.91	88.68	27

## Data Availability

The data are available upon request.
